# Continuing use of e‐cigarettes after stopping smoking and relapse: Secondary analysis of a large randomised controlled trial

**DOI:** 10.1111/add.70294

**Published:** 2026-01-21

**Authors:** Peter Hajek, Dunja Przulj, Katie Myers Smith, Jinshuo Li, Peter Sasieni, Louise Ross, Hayden McRobbie, Maciej Goniewicz, Francesca Pesola

**Affiliations:** ^1^ Wolfson Institute of Population Health Queen Mary University of London London UK; ^2^ Department of Health Sciences University of York York UK; ^3^ National Centre for Smoking Cessation and Training Leicester UK; ^4^ National Drug and Alcohol Research Centre University of New South Wales Sydney Australia; ^5^ Roswell Park Comprehensive Cancer Center Buffalo NY USA

**Keywords:** e‐cigarette, NRT, RCT, relapse, secondary analysis, vaping

## Abstract

**Background and aims:**

Smokers quitting successfully with the help of e‐cigarettes often continue vaping. It is not known whether this promotes or prevents relapse back to smoking. This study aimed to determine whether use of e‐cigarettes after successful smoking cessation affects the probability of relapse later on.

**Design:**

Secondary analysis of a randomised controlled trial where participants received combination nicotine replacement therapy (NRT) or e‐cigarettes to compare relapse rates in the two study arms and in abstainers who did and did not use e‐cigarettes.

**Setting:**

Four stop‐smoking services in the United Kingdom.

**Participants:**

886 smokers (median age 41, smoking on average 15 cigarettes per day, 48% female) seeking help with stopping smoking.

**Measurements:**

Main outcome was relapse to smoking by 12 months in participants who were abstinent at 4 weeks or at 6 months. Relapse was defined as abstinence at 4 weeks but not at one year or abstinence at 6 months but not at one year. Abstinence from smoking was defined as no smoking over the past 7 days. E‐cigarette use was defined as using e‐cigarettes at the time of abstinence on at least one day per week.

**Findings:**

Abstainers in the e‐cigarette arm were less likely to relapse than abstainers in the NRT arm [relative risk (RR) = 0.78, 95% confidence interval (CI) = 0.64–0.96 for relapse between 4 weeks and 1 year; RR = 0.71, 95% CI = 0.55–0.93 for relapse between 6 months and 1 year). Relapse rates over both time periods were also lower in abstainers who used e‐cigarettes compared with abstainers who did not use e‐cigarettes (RR = 0.79, 95% CI = 0.65–0.97 and RR = 0.75, 95% CI = 0.57–0.98, respectively).

**Conclusions:**

Use of e‐cigarettes after stopping smoking is associated with a reduced risk of relapse.

## INTRODUCTION

E‐cigarettes (EC) are more effective in helping smokers quit than other types of nicotine replacement therapy (NRT) [1]. This is most likely because, compared with licensed NRT products, EC provide nicotine delivery and sensory input that are closer to what smokers obtain from cigarettes [2]. As a consequence, smokers who stop smoking with the help of EC are more likely to continue using them than is the case with NRT [3].

It is currently not clear whether such use should be discouraged. EC use is likely to carry some health risks, but these are expected to be only a small fraction of the risks of smoking [4]. The key question is therefore whether the use of EC post‐smoking cessation decreases or increases the risk of relapse back to smoking. If EC use protects ex‐smokers from relapse, it would have a positive health impact, but if it increases the relapse risk, ex‐smokers should be advised to stop using EC as well.

An earlier meta‐analysis of several cohort studies reported a higher relapse rate in EC users than in non‐users [5], but this could be associated with EC users quitting more recently (i.e. non‐users included people who had stopped smoking before EC became available). Quitters who did not need aids may have also been less dependent than those needing EC. A newer review of this literature reported insufficient control for confounding variables and inconsistent results [6].

Data from trials evaluating EC efficacy can be more informative than cohort studies. This is because they avoid the problem with the unequal duration of abstinence and also, to an extent, the key self‐selection issue of dependence, as all participants seek help with stopping smoking. An attempt to derive relapse rates from changes in abstinence rates in existing randomised trials reported mixed findings that were difficult to interpret [7]. No study so far compared relapse rates in participants who did and did not use EC, regardless of study arm allocation.

Two studies pointed to the importance of the definition of abstinence. Using sustained abstinence, where temporary lapses are classified as relapse, even if abstinence is regained, leads to different results than using point‐prevalence abstinence, where past lapses are allowed [7, 8]. Only point prevalence abstinence allows an assessment of whether EC use affects the chance that lapses trigger a full relapse.

We conducted a secondary analysis of data from a large, randomised trial comparing EC and NRT [9], and looked at relapse rates in the two study arms as well as in quitters who did and did not use EC. In the parent trial, we used sustained abstinence, validated at 4 weeks and at 12 months, and reported that relapse between 4 weeks and 12 months was higher in the NRT arm, but not significantly so (RR = 1.27, 95% CI = 0.93–1.73). In the current secondary analysis we used point prevalence rather than sustained abstinence, and we also examined whether any effects on relapse unfolded early on, between 4 weeks and 6 months, or later, between 6 months and 1 year.

## METHODS

### Design

The parent trial for this secondary analysis was the Trial of Electronic Cigarettes (TEC; registration ISRCTN60477608) [9]. In brief, the trial included 886 smokers randomised to receive a combination of NRT products of their choice, supplied for up to 3 months, or an EC starter pack comprising a refillable EC device (The UK Ecig Store OneKit) and one 30 ml bottle of 18 mg/ml nicotine e‐liquid (expected to last for about 2 weeks), with instructions to buy further supplies with strength and flavours of their choice themselves. The 1‐year sustained biochemically validated abstinence from smoking was 18.0% in the EC group and 9.9% in the NRT group, with EC also being more cost‐effective than NRT [10].

### Measures

When this report mentions abstinence or relapse, this means abstinence from or relapse to smoking.

Abstinence was defined as self‐reporting smoking not a puff in the previous 7 days (7‐day point‐prevalence abstinence). This means that abstinence did not have to be sustained, e.g. participants could be classified as abstinent at 6 months even if they were not abstinent at 4 weeks. Point prevalence rather than sustained abstinence was used to capture any recovery from lapses. We also used self‐reported rather than validated abstinence because only sustained (not point‐prevalence) abstinence was validated at 1 year, and we did not verify abstinence biochemically at 6 months.

Relapse at 1 year was defined as abstinence at 4 weeks but not at 1 year or abstinence at 6 months but not at 1 year.

Regarding EC use, participants who reported using EC at the time of abstinence (e.g. abstinent and using EC at 4 weeks or at 6 months) on at least 1 day per week were classified as EC users (i.e. weekly use).

### Statistical analysis

We examined whether allocation arm (EC vs NRT) and using EC (vs not using EC, regardless of study arm allocation) were associated with relapse. We used a generalised linear model (GLM) with family set as binomial with a log link to estimate risk ratios and 95% confidence intervals. If the model failed to converge, we specified the family as Poisson with log link and robust standard errors. Analyses were adjusted for variables associated with abstinence at 12 months (age, sex and Fagerström Test for Cigarette Dependence) and for study site, a randomisation stratifier in the parent trial. Participants who died (one in each arm) were excluded, reducing *n* from 886 to 884.

The analyses are exploratory and were conducted using Stata 18.0 (StataCorp LLC, College Station, TX, USA).

## RESULTS

Sample characteristics were reported in the parent trial [9]. In brief, the median age of the sample was 41 years, 48% were women, 69.6% were in paid employment, and they smoked on average 15 cigarettes per day and their baseline expired air carbon monoxide reading was 20 ppm. There were no differences between participants in the two study arms.

Abstainers in the EC arm were less likely to relapse back to smoking than abstainers in the NRT arm, both between 4 weeks and 12 months (RR = 0.78, 95% CI = 0.64–0.96) and between 6 months and 12 months (RR = 0.71, 95% CI = 0.55–0.93). For more details, see Table 1.

**TABLE 1:** Relapse rates in the two study arms.

	Abstinent at 4 weeks *n*	Relapsed at 12 months *n* (%)	Abstinent at 6 months *n*	Relapsed at 12 months *n* (%)
**EC arm, *n* = 438**	195	94 (48.2)	157	59 (37.6)
**NRT arm, *n* = 446**	136	83 (61.0)	115	60 (52.2)
**RR (95% CI)** ^**a**^		RR = 0.78 (0.64–0.96)		RR = 0.71 (0.55–0.93)

Abbreviations: EC = electronic cigarette; NRT = nicotine replacement therapy.

^a^ Relative risk (95% CI) estimated by regressing relapse onto arm (EC vs NRT), while adjusting for age, sex and Fagerström Test for Cigarette Dependence.

Abstainers who used EC had lower relapse rates than those who did not use EC between 4 weeks and 12 months (RR = 0.79, 95% CI = 0.65–0.97) and between 6 months and 12 months (RR = 0.75, 95% CI = 0.57–0.98). For more details, see Table 2.

**TABLE 2:** Relapse rate in abstainers who did and did not use EC.

	Abstinent at 4 weeks *n*	Relapsed at 12 months *n* (%)	Abstinent at 6 months *n*	Relapsed at 12 months *n* (%)
**Using EC** ***n* = 357 at 4 weeks** ***n* = 364 at 6 months**	199	97 (48.7)	162	62 (38.3)
**Not using EC** ***n* = 527 at 4 weeks** ***n* = 520 at 6 months**	132	80 (60.6)	110	57 (51.8)
**RR (95% CI)** ^**a**^		RR = 0.79 (0.65–0.97)		RR = 0.75 (0.57–0.98)

Abbreviations: EC = electronic cigarettes.

^a^ Relative risk (95% CI) estimated by regressing relapse onto EC use status (use vs no use), while adjusting for age, sex and Fagerström Test for Cigarette Dependence.

Tables S1 and S2 show further breakdowns of relapse rates and EC use at different time points.

## DISCUSSION

In this trial, relapse rates between 4 weeks and 1 year, as well as between 6 months and 1 year, were lower in the study arm assigned to EC than in the study arm assigned to NRT. Relapse rates over both time periods were also lower in abstainers who used EC compared with abstainers who did not use EC.

The size of the effect is similar in the two comparisons, because most abstainers in the EC arm were using EC. The finding of lower relapse rates in abstainers who used EC compared with abstainers who did not use them does not necessarily show a causal effect because it could have been affected by self‐selection. However, the finding concerning randomised groups suggests causality.

The study has limitations. Abstinence was not biochemically verified and so it could have been overestimated. Future trials of EC should validate self‐reported abstinence in all abstainers, not just those with sustained abstinence, and report relapse rates between early and late follow‐ups using point‐prevalence abstinence. The length of the follow‐up was 1 year. Further data are needed to assess whether the findings hold for longer time periods and apply to EC use that is more prolonged.

In summary, the current study provides the first evidence that smokers who successfully stop smoking and continue EC use reduce their risk of relapse back to smoking, but further studies are needed to verify this finding. If replicated, the finding would provide evidence that successful quitters who continue to use EC should not be discouraged from such use.

## AUTHORS CONTRIBUTIONS

**Peter Hajek**: Conceptualisation; funding acquisition; methodology; supervision; writing—original draft; writing—review and editing. **Dunja Przulj**: Conceptualisation; investigation; validation; writing—original draft; writing—review and editing. **Katie Myers Smith**: Conceptualisation, investigation, project administration, validation, writing—original draft; writing—review and editing. **Jinshuo Li**: Conceptualisation; writing—review and editing. **Peter Sasieni**: Conceptualisation; writing—review and editing. **Louise Ross**: Conceptualisation; writing—review and editing. **Hayden McRobbie**: Conceptualisation; methodology; writing—original draft; writing—review and editing. **Maciej Goniewicz**: Conceptualisation; writing—review and editing. **Francesca Pesola**: Conceptualisation, data curation, formal analysis, methodology, writing—original draft; writing—review and editing.

## DECLARATION OF INTERESTS

L.R. received a speaker fee in 2024 from Consilient Health.

## CLINICAL TRIAL REGISTRATION

ISRCTN60477608 (https://www.isrctn.com/ISRCTN60477608).

## Supporting information


**Table S1.** EC use and relapse in the two study arms.
**Table S2.** EC use at 12 months and relapse.

## Data Availability

The data that support the findings of this study are available from the corresponding author, upon reasonable request.
