# Supplementary material for: Continuing use of e‐cigarettes after stopping smoking and relapse: Secondary analysis of a large randomised controlled trial
Source: Addiction. 2026 Jan 21;121(4):994–7. doi: 10.1111/add.70294 (PMC12980290; doi:10.1111/add.70294)
Supplement: Supplementary file 1 — Table S1. EC use and relapse in the two study arms. Table S2. EC use at 12 months and relapse. [file ADD-121-994-s001.docx]

**Supplementary Table 1. EC use and relapse in the two study arms**

|  | **Abstinent at 4-weeks**  **N** | **Relapsed at 12-month**  **N (%)** | **Abstinent at**  **6-months**  **N** | **Relapsed at 12-months**  **N (%)** |
| --- | --- | --- | --- | --- |
| **EC arm using EC** | 193 | 93 (48.2) | 131 | 46 (35.1) |
| **EC arm not using EC** | 2 | 1 (50) | 26 | 13 (50) |
| **NRT arm using EC** | 6 | 4 (66.7) | 31 | 16 (51.6) |
| **NRT arm not using EC** | 130 | 79 (60.8) | 84 | 44 (53.4) |
| **Total** | 331 | 177 | 272 | 119 |

Abbreviations: EC – electronic cigarettes; NRT – nicotine replacement therapy

**Supplementary Table 2. EC use at 12 months and relapse**

|  | **Did not relapse between 4 weeks and 1 year** | | **Relapsed between**  **4 weeks and 1 year** | |  |
| --- | --- | --- | --- | --- | --- |
|  | **N** | **Using EC at 12 months: N (%)** | **N** | **Using EC at 12 months: N (%)** | **RR (95%CI)^** |
| **EC Arm** | 101 | 90 (89.1) | 94 | 39 (41.5) | 0.36 (0.27-0.49) |
| **NRT arm** | 53 | 16 (30.2) | 83 | 15 (18.1) | 0.70 (0.47-1.04) |

Abbreviations: EC – electronic cigarettes; NRT – nicotine replacement therapy

^ RR (95%CI) estimated by regressing relapse onto EC use status (use vs. no use), while adjusting for age, sex, and Fagerstrom Test for Cigarette Dependence for EC and NRT arm separately
